# Combined treatment of rituximab, idarubicin, dexamethasone, cytarabine, methotrexate with radiotherapy for primary central nervous system lymphoma

**DOI:** 10.1111/jcmm.12252

**Published:** 2014-03-13

**Authors:** Defeng Zhao, Liren Qian, Jianliang Shen, Xiaopeng Liu, Ke Mei, Jian Cen, Yaming Wang, Congyong Li, Yuanyuan Ma

**Affiliations:** a Department of Hematology, Navy General Hospital of PLABeijing, China; b Department of Radiology, Shanghai Jiaotong University Affiliated First People's HospitalShanghai, China; c Department of Neurosurgery, Navy General Hospital of PLABeijing, China; d Department of Gastroenterology, Cadre Ward, Navy General Hospital of PLABeijing, China

**Keywords:** R-IDARAM, primary central nervous system lymphoma, diffuse large B-cell lymphoma, stereotactic brachytherapy

## Abstract

The overall response rates and long-term survival of primary central nervous system lymphoma (PCNSL) are still significantly inferior to the results achieved in similar subtypes of extranodal non-Hodgkin's lymphoma. It is clearly necessary to investigate new therapeutic methods on PCNSL. We encountered three patients histologically documented PCNSL as diffuse large B-cell lymphoma (DLBCL). They were treated with R-IDARAM which comprised rituximab, idarubicin, dexamethasone, cytarabine and methotrexate. Patient 1 received stereotactic brachytherapy (SBT) prior to chemotherapy performed with iodine-125 seeds (cumulative therapeutic dose 50 Gy). After six cycles of R-IDARAM at 3-weekly intervals, radiotherapy was applied at a dosage of 2000–4000 cGy in conventional schedule (180 or 200 cGy/day) to whole brain or spinal cord in all patients. Complete remission (CR) was achieved after first two cycles of R-IDARAM in all patients. All three patients remained in CR at the time of this report with a median duration of follow-up of 23 months (ranging from 13 to 41 months). Three patients have been alive for 41, 13, 16 months respectively until now. The patient with the longest survival time was the one given SBT prior to chemotherapy. This study suggests that R-IDARAM combining with radiotherapy maybe a high effective regimen in PCNSL patients especially those with primary central nervous system DLBCL. A comprehensive treatment combining internal radiotherapy by SBT, modified R-IDARAM and followed reduced external radiotherapy may be a new treatment concept for PCNSL with higher efficiency and lower toxicity.

## Introduction

Primary central nervous system lymphoma (PCNSL) is a rare subtype of non-Hodgkin's lymphoma (NHL) that is confined to the brain, eyes, leptomeninges or spinal cord in the absence of extracerebral tumour manifestation and metastases [1]. Primary central nervous system lymphoma accounts for 3.3% of all brain tumours [1,2]. Diffuse large B-cell lymphoma (DLBCL) accounts for about 90% of PCNSL according to the World Health Organization (WHO) Lymphoma Classification. The remainders include Burkitt's lymphoma, T-cell-rich B-cell lymphoma, peripheral T-cell lymphoma and rarely ‘low-grade’ B-cell lymphoma [3,4]. Overall incidence of PCNSL in the immunocompetent population has been increasing during the last several decades, and it is one of the few malignant primary brain tumours that is sensitive to both chemotherapy and radiotherapy treatment.

Because of methotrexate (MTX)-based combination chemotherapy, survival has improved over the last three decades. The addition of MTX-based combination chemotherapy to whole brain radiotherapy has improved disease-free survival and overall survival significantly [5–7]. But the overall response rates and long-term survival are still significantly inferior to the results achieved in similar subtypes of extranodal NHL [8]. Long-term treatment-related neurological toxicity remains a major problem. It is clearly necessary to investigate new therapeutic methods on PCNSL.

Stereotactic brachytherapy (SBT) with implantation of iodine-125 seeds is a kind of well-developed internal radiotherapy method which has been successfully used in certain CNS tumours with many benefits such as few side effects, continuously killing tumour cells, *etc*., but it has not been used in CNS lymphoma previously [9,10]. Stereotactic brachytherapy with implantation of iridium-192 sources was applied on PCNSL as Apuzzo *et al*. described [11] in 1980s, but it has not been widely used. To our knowledge, SBT with implantation of iodine-125 seeds was given prior to chemotherapy to one of the patients for the first time in this study.

R-IDARAM chemotherapy regimen (rituximab, idarubicin, dexamethasone, cytosine arabinoside, MTX, intrathechal MTX and cytosine arabinoside) was applied in very few patients until now [2]. In this study, we modified the regimens by reducing MTX to 2 g/m^2^ to reduce its toxicity and rituximab was used independently on day 1 because of the long time of infusion. Herein, we report one PCNSL patient received a comprehensive treatment with SBT, modified R-IDARAM, and followed reduced external radiotherapy and the other two patients presenting with mass lesions in the CNS, received specifically CNS targeted chemotherapy in combination with consolidated external radiotherapy.

## Methods and patients

This study was performed at departments of Haematology and Neurosurgery in Navy General Hospital in China between August 2010 and November 2013. We have encountered three patients with histologically documented CNSL. Informed consent was provided by each patient. Patient 1 and patient 2 were newly diagnosed as primary central nervous system DLBCL by stereotactic biopsy in the department of Neurosurgery as previously described [12,13]. Patient 2 was newly diagnosed as primary central nervous system DLBCL by CT-guided aspiration biopsy. The modified R-IDARAM chemotherapy regimen was applied to all patients. This regimen comprised of rituximab 375 mg/m^2^ (day 1); idarubicin 10 mg/m^2^ (day 2 and 3); dexamethasone 100 mg/m^2^ (12 hrs infusion in day 2, 3 and 4); cytarabine 1 g/m^2^ (1 hr infusion in day 2 and 3); MTX 2 g/m^2^ (6 hrs infusion in day 4 with folinic acid rescue) and cytarabine 70 mg plus 12 mg MTX *via* intrathecal route in day 2 and 9. Colony-stimulating factor (150 μg/m^2^) was also started at the seventh day of chemotherapy. Chemotherapy cycles were given at 3-weekly intervals. After course 6, external radiotherapy was applied to whole brain or spinal cord at a dosage of 2000–4000 cGy in conventional schedule (180 cGy or 200 cGy per day). However, in patient 1, SBT was applied when biopsy was being carried out by using iodine-125 seeds (cumulative therapeutic dose 50 Gy) prior to chemotherapy as previously described [9,14,15]. Chemotherapy was performed after SBT.

Response to chemotherapy and the toxicity were evaluated every two courses of chemotherapy and after external RT according to Response Criteria by Lauren E. Abrey *et al*. [8] ([Table1). Patients who achieved complete remission (CR) were followed up every 3 months in the first year. At follow-up visits, complete neurological and ophthalmological examination, and MRIs were performed. The toxicity of the regimen was graded by objective measures according to the Common Toxicity Criteria [16].

**Table 1:** Response criteria for primary central nervous system lymphoma 8

Response	Brain imaging	Corticosteroid dose	Eye examination	CSF cytology
CR	No contrast enhancement	None	Normal	Negative
CRu	No contrast enhancement	Any	Normal	Negative
	Minimal abnormality	Any	Minor RPE abnormality	Negative
PR	50% decrease in enhancing tumour	Irrelevant	Minor RPE abnormality or normal	Negative
	No contrast enhancement	Irrelevant	Decrease in vitreous cells or retinal infiltrate	Persistent or suspicious
PD	25% increase in lesion	Irrelevant	Recurrent or new ocular disease	Recurrent or positive
	Any new site of disease: CNS or systemic			

CR, complete response;

CRu, unconfirmed complete response;

RPE, retinal pigment epithelium;

PR, partial response;

PD, progressive disease.

### Patient 1

A 57-year-old man was admitted to the hospital with half a month history of right-side limb inflexible and fatigue. A mild reduced muscle strength on right-side limb was detected (Level IV) by clinical examination. MR imaging studies showed a mass lesion on the left basal ganglia. A tumour biopsy was obtained by stereotactic operation. Stereotactic brachytherapy was performed at the same time. Histopathological examination showed DLBCL. Immunohistochemical examination revealed CD20(+++), LCA(+++), CD79(++), MuM-1(++), BCL-2(+), CD39(−), CD45RO(−), BCL-6(−), CD10(−), GFAP(−), SYN(−), S-100(−), CD68(−), NeuN(−), CD34(−), EMA(−), Olig-2(−), NF(−), WT-1(−) and Ki-67 index 90%.

### Patient 2

A 49-year-old woman was admitted to the hospital with a month history of irrelevant answers and slow responsive. Clinical examination was negative. MR imaging studies showed multiple mass lesions on bilateral lobi temporalis and the left frontal lobe. A tumour biopsy was obtained by stereotactic operation. Histopathological examination showed DLBCL. Immunohistochemical examination revealed LCA(+++), CD20(+++), PAX5(+++), CD3(−), CD5(−), FOXP1(+++), BCL2(++),BCL6(++), MuM-1(++), CD10(−), ALK(−), TDT(−), OLig2(−), GFAP(−),CD138(−), CD30(−) and Ki-67 index >90%.

### Patient 3

A 53-year-old man was admitted to the hospital with half a month history of dysuresia and fatigue. Right-side hemiplegic paralysis and reduced muscle strengths were detected on the left upper limb muscle (Level IV) and the left lower limb muscle strength (Level I) by clinical examination. MR imaging studies showed a mass lesion on the lumbar spinal canal. A tumour biopsy was obtained *via* CT-guided aspiration biopsy. Histopathological examination showed DLBCL. Immunohistochemical examination revealed LCA(+++), Vimentin(+++), AE1/AE3(−), CD20(+++), CD79a(++), CD3(−), TdT(−), Bcl-6(+), CD10(+), MuM-1(−), CD138(−), Bcl-2(−), CD43(+), HHV-8(−) and Ki-67 index >95%.

## Results

The mean age of three patients was 53 (range 49–57). Clinical and radiological features of patients are summarized in Table2. The time between the onset of the symptoms and admission to the hospital were 0.5–1 month. In all patients HIV, HBV and anti-HCV antibodies were negative. In all patients, the tumours were diagnosed as DLBCL according to the revised European-American classification of lymphoid neoplasms (REAL) and to the WHO Classification of neoplastic diseases of the haematopoietic lymphoid tissues [17].

**Table 2:** Clinical and radiological features of patients with PCNSL

Case No.	1	2	3
Age	57	49	53
Sex	Male	Female	Male
B symptoms	−	−	+
Other symptoms	Right-side limb inflexible, fatigue	Irrelevant answers, slow responsive, right-side hemiplegic paralysis	Dysuresia, fatigue
Lymphadenopathy	−	−	−
Bone marrow involvement	−	−	−
Number of mass lesion	Single	Multiple	Single
Size (cm)	3.5 × 4.5	3.8 × 5.1 (maximal)	1.3 × 3.5
LDH	102 U/l (Normal)	151 U/l (Normal)	106 U/l (Normal)
KPS	50	60	10
IPI	3	2	2
Response	CR	CR	CR
Survival (months)	41	13	16

B symptoms: fever, night sweats and weight loss;

LDH: lactate dehydrogenase;

KPS: Karnofsky performance score;

PCNSL: primary central nervous system lymphoma.

In all three patients, CR was achieved after two chemotherapy cycles of R-IDARAM (Figs 3). All three patients remained in CR at the time of this report with a median duration of follow-up of 23 months (range 13–41 months). Three patients have been alive for 41, 13, 16 months respectively until now. The patient with the longest survival time was the one given SBT prior to chemotherapy.

**Figure 1:**
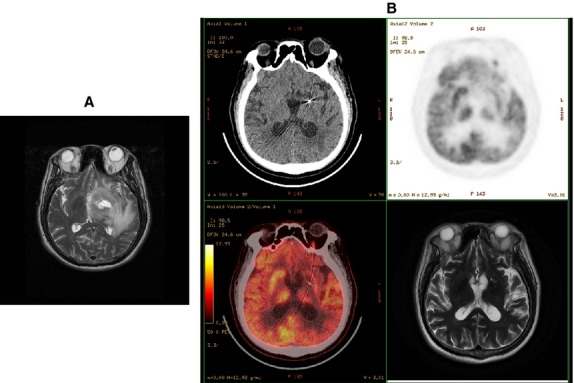
In Patient 1, MR scan shows a mass lesion on the left basal ganglia (A). After stereotactic brachytherapy, chemotherapy and reduced external RT, CT/PET-CT/MR images show the lesion disappeared (B).

**Figure 2:**
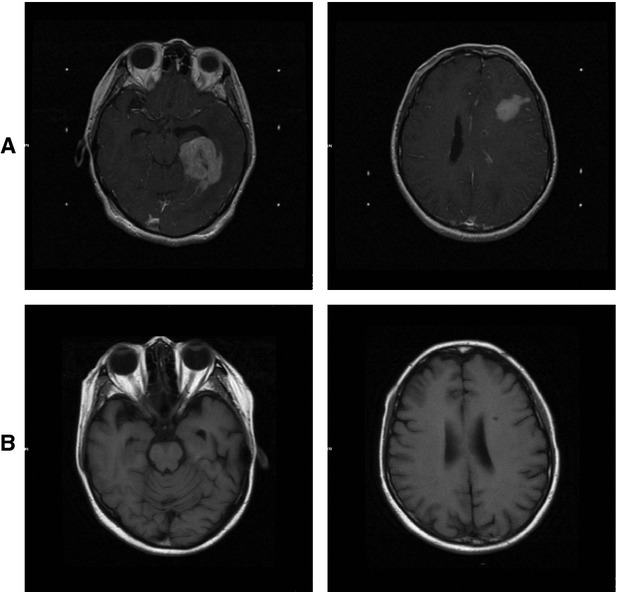
In Patient 2, MR scan shows multiple mass lesions on bilateral lobi temporalis and the left frontal lobe (A). After two cycles of chemotherapy, MR images show the lesion disappeared (B).

**Figure 3:**
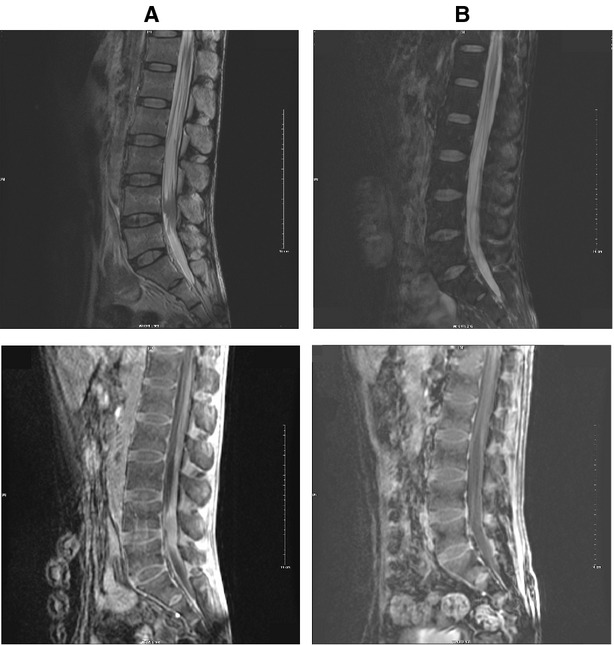
In Patient 3, MR scan shows a mass lesion on the lumbar spinal canal (A). After two cycles of chemotherapy, MR images show the lesion disappeared (B).

Observed acute chemotherapy-related toxicities were shown in Table3. All these medical problems were resolved with supportive medical treatments. Toxicity depending on RT was not seen except dermal and mucosal toxicities during RT until now. All these symptoms disappeared with symptomatic treatments.

**Table 3:** Acute chemotherapy-related toxicity

Toxicity/grade (ECOG criteria)	Patient 1	Patient 2	Patient 3
Hematologic	3	0	0
Infection	2	1	1
Fever in absence of infection	2	1	1
Mucosal	1	0	0
Nausea and vomiting	2	1	1
Renal/bladder	0	0	0
Neurologic	1	0	0
Cardiac	0	0	0
Liver	2	1	0
Gastrointestinal	0	0	0

## Discussion

Although regimens such as R-MPV (rituximab, MTX, vincristine, procarbazine), MBVP (MTX, teniposide, carmustine and methylprednisolone), CHOD/BVAM (cyclophosphamide, doxorubicin, vincristine, dexamethasone/vincristine, cytosine arabinoside, MTX) *etc*. have shown promise [18–20], there is a clear need to continue to try to optimize chemotherapy regimens and to explore new comprehensive treatments on PCNSL because of lower 2-year overall and progression-free survival than the results achieved in similar subtypes of extranodal NHL, limited doses of chemotherapy deliverable into the CNS, high rates of neurotoxicity, *etc*. [2,18]. To our knowledge, combining SBT, chemotherapy and reduced external radiotherapy as a novel multimodal treatment concept was firstly used in PCNSL in this study which showed great efficiency and safety.

Chemotherapy was the first-line treatment for PCNSL if patients are sufficiently fit. The IDARAM regimen was found to be effective in seven patients with PCNSL by Moreton *et al*. [2]. and was modified by Yilmaz *et al*. to R-IDARAM in three patients with PCNSL [21]. The most important problem of drug deliver is the blood–brain barrier. To achieve therapeutic concentrations of MTX in the brain, high doses are required (*i.e*. at least 1.5 g/m^2^). Cytarabine can penetrate to the brain parenchyma and cerebrospinal fluid (CSF) ara-c concentrations ranged from 1.2 μM at 4 g to 4.1 μM at 18 g as Donehower *et al*. reported [22]. Most anthracyclines probably do not cross the blood–brain barrier. But as the major circulating metabolite of idarubicin, idarubicinol is present in the CSF following intravenous administration idarubicin at sustained levels [23]. Dexamethasone will also penetrate the blood–brain barrier [24]. Rituximab has been detected in the CSF at concentration at most 0.1% after intravenous administration in patients with CNS lymphoma [25]. Rituximab transport to the CSF may occur *via* leaking across areas of blood–brain barrier breakdown in the lymphoma and/or macromolecular vesicular transport of the antibody across an intact blood–brain barrier [26]. The R-IDARAM was still not widely used in PCNSL with good efficiency and tolerable toxicity. In this study, we modified the regimens by reducing MTX to 2 g/m^2^ to reduce its toxicity without reducing its efficiency.

In this study, consolidation radiotherapy is performed in all three patients after chemotherapy. There is evidence that the addition of radiotherapy can achieve modest improvement in disease-free and overall survival [27]. The role of consolidation radiotherapy is controversial because of limited long-term efficiency [28] and neurotoxicity, which presents as dementia, ataxia and urinary incontinence, and is associated with MRI evidence of leucoencephalopathy after a delay of several years [29,30]. The risk of delayed neurotoxicity is generally thought to be the greatest in elderly patients [6].

Although in these three patients, neurotoxicity of radiotherapy was not seen currently because of the short-term follow-up, it is not assured whether neurotoxicity will appear in the following years. In this study, patient 1 has survived for 41 months with disease-free until now who was performed SBT before chemotherapy and reduced external RT after chemotherapy.

Stereotactic brachytherapy with implantation of iodine-125 seeds represents a safe and effective local treatment option for certain brain tumours [31–33]. Histological evaluation within the same operative session, precise treatment of the tumour, maximal sparing of surrounding healthy tissue, minimal rate of long-term complications and preservation of the whole therapeutic spectrumin case of tumour progression (*e.g*. reimplantation, external radiotherapy) are described hallmarks of SBT [32–34].

In this study, we assessed the feasibility of SBT as a local treatment combination with following chemotherapy and consolidation external radiotherapy for a new try. Consolidation external radiotherapy was performed with a much lower dose than the other two patients which may also reduce the related neurotoxicity of radiation. This kind of comprehensive treatment may be a new concept to treat PCNSL and other CNS tumours with higher efficiency and lower toxicity. Such an approach will be the basis for further study.

## Conclusions

We believe that modified R-IDARAM consolidated with radiotherapy may improve disease control and outcome of patients. A comprehensive treatment combining SBT, modified R-IDARAM and followed reduced external radiotherapy may be a novel multimodal treatment concept to treating PCNSL with higher efficiency and lower toxicity which is valuable for multicentre clinical trials to investigate in the future.
